# Real-Time Polymerase Chain Reaction for Detecting SARS Coronavirus, Beijing, 2003

**DOI:** 10.3201/eid1002.030799

**Published:** 2004-02

**Authors:** Junhui Zhai, Thomas Briese, Erhei Dai, Xiaoyi Wang, Xin Pang, Zongmin Du, Haihong Liu, Jin Wang, Hongxia Wang, Zhaobiao Guo, Zeliang Chen, Lingxiao Jiang, Dongsheng Zhou, Yanping Han, Omar Jabado, Gustavo Palacios, W. Ian Lipkin, Ruifu Yang

**Affiliations:** * Institute of Microbiology and Epidemiology, Beijing, China; † Mailman School of Public Health of Columbia University, New York, New York, USA; 1 Junhui Zhai and Thomas Briese contributed equally to the manuscript.

**Keywords:** SARS-CoV, PCR, diagnostic

## Abstract

During the 2003 severe acute respiratory syndrome (SARS) outbreak, a real-time quantitative polymerase chain reaction, which targets the nucleocapsid gene at the 3′-end of the viral genome, was established to detect and identify the SARS-associated coronavirus. We describe the use of this assay to screen >700 clinical samples.

Severe acute respiratory syndrome (SARS) is a new infectious disease of humans, first recognized in late February 2003 in Hanoi, Vietnam. The disease spread rapidly, with cases reported from 29 countries on five continents over 4 months (*1*–*7*). By July 3, 2003, this epidemic resulted in 8,439 reported cases globally, of which 812 were fatal (*8*). Rapid identification of the causal agent as a novel coronavirus (SARS-CoV) represents an extraordinary achievement in the history of global health and helped to contain the epidemic (*7*). Nonetheless, the epidemiology and pathogenesis of SARS remain poorly understood, and definitive diagnostic tests or specific treatments are not established. Since the origin of the virus and its animal reservoirs remain to be defined, the potential for recurrence is unknown. This fact underscores the importance of establishing sensitive and efficient methods for diagnosis and surveillance.

Immunofluorescence and enzyme-linked immunosorbent assays (ELISA) are reported to inconsistently detect antibodies to SARS-CoV before day 10 or 20 after the onset of symptoms, respectively (*7*,*9*). Thus, although helpful in tracking the course of infection at the population level, these serologic tools have less usefulness in detecting infection at early stages, when there may be potential to implement therapeutic interventions or measures, such as quarantine that may reduce the risk for transmission to naïve persons. In contrast, polymerase chain reaction (PCR)–based assays have the potential to detect infection at earlier time points. We describe a sensitive real-time PCR assay that can be readily standardized across laboratories and report its use in a survey of more than 700 samples from persons diagnosed with probable SARS during the 2003 epidemic in Beijing.

## The Study

Primers and probe were selected in the N (nucleocapsid protein) gene region at the 3′ end of the SARS-CoV genome by using Primer Express Software (PE Applied Biosystems, Foster City, CA). The primer set used was: Taq-772F 5′-AAGCCTCGCCAAAAACGTAC (forward) and Taq-1000R 5′-AAGTCAGCCATGTTCCCGAA (reverse), Taq-955T 5′-FAM-TCACGCATTGGCATGGAAGTCACAC-T-TAMRA (probe), labeled with the reporter FAM (6-carboxyfluorescein) and the quencher TAMRA (6-carboxytetramethylrhodamine) (TIB Molbiol, Berlin, Germany).

A calibration standard was generated by PCR amplification of a 1,277–bp fragment comprising part of the N open reading frame (ORF) and the 3′ noncoding region (Co-STND-U275, 5′-CCCGACGAGTTCGTGGTGGTG; Co-STND-L1529, 5′-GCGTTACACATTAGGGCTCTTCCATA). The product was cloned into vector pGEM-Teasy (Invitrogen, Carlsbad, CA), and serial dilutions of linearized plasmid were used to optimize the assay. RNA standards were generated by in vitro transcription of linearized plasmid DNA using a mMESSAGE mMACHINE T7 kit as recommended by the manufacturer (Ambion, Austin, TX). A portion of the construct (nucleotides 682–1105 of the N ORF) was modified through site-directed mutagenesis, to distinguish plasmid-derived products from authentic products in diagnostic applications. Mutations introduced were an A to G change at position 845 of the N ORF, and an A to C change at position 866, creating a unique *Apa*I restriction site.

Detection of live virus was assessed by using supernatant from virus-infected Vero E6 cells (isolate BJ01; 4th passage; 10^8^ TCID_50_/mL) tenfold diluted to 10^–12^ in tissue culture media. RNA from 140-μL aliquots of each dilution was extracted and resuspended in 60 μL of DEPC-treated water for reverse transcription (9 μL RNA/20-μL reaction) and PCR (5 μL/assay). 20 μL of each virus dilution were spiked into 180 μL of clarified supernatant of a fecal preparation to simulate clinical specimens, and RNA from 140-μL aliquots was extracted and processed as above.

Clinical materials, including 326 fecal and 426 whole blood samples, were collected from Chaoyang Hospital, 301 Hospital, You’an Hospital, and Xuanwu Hospital, Beijing. All persons had a diagnosis of probable SARS according to World Health Organization (WHO) criteria. For analysis of fecal samples, 1 g of stool was suspended in 1 mL of phosphate-buffered saline, mixed vigorously, and centrifuged for 10 min at 3,000 *g*, 4°C. Supernatant was collected for RNA extraction and PCR analysis. For analysis of blood samples, whole blood was fractionated using Ficoll Paque (Amersham Pharmacia, England). Plasma was collected and immunoglobulin (Ig) G and IgM levels were determined with an ELISA kit from the Beijing Genomics Institute (Beijing, China). Peripheral blood mononuclear cells were collected and RNA extracted by using the QiaAmp Viral RNA Mini Kit (Qiagen, Germany). Nine microliters total RNA was reverse transcribed (SuperScript II Transcriptase, Invitrogen), and 2 μL of cDNA subjected to PCR by using a TaqMan Universal Master Mix kit (PE Applied Biosystems) on an ABI Prism 7900 HT sequence detector (PE Applied Biosystems). Thermocycling conditions were: 2 min 50°C (AmpErase UNG), 10 min 95°C (polymerase activation); 45 cycles of 15s 95°C denaturation, and 1 min 60°C annealing/extension.

## Conclusions

**Figure 1:**
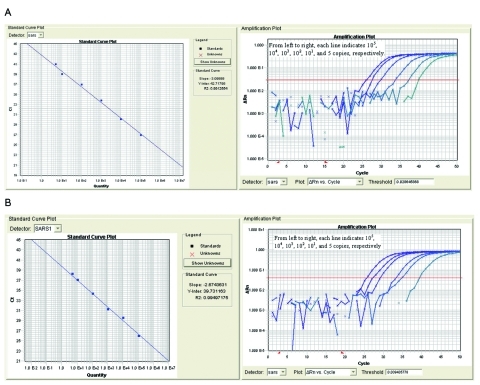
Standard curve and amplification plot using serial dilutions of plasmid DNA (A) or of cRNA (B).

A standard curve of plasmid concentration versus threshold cycle was generated with a cloned version of the 3′ terminal portion of the viral genome. A correlation coefficient (r2) of 0.9913 showed a linear relationship between threshold cycle (Ct) and plasmid concentration (0–10^5^ copies) (Figure 1A). The detection limit for plasmid DNA was <5 copies per assay (Ct = 42.66). A linear relationship was consistently obtained for input loads of 10^1^–10^5^ copies per assay.

Standards for RT-PCR were generated by in vitro transcription of RNA from linearized plasmid template with T7 polymerase. Logarithmic dilutions of the synthesized RNA yielded results comparable to the DNA standards (r2 = 0.9950; Figure 1B).

Supernatant from infected Vero E6 cells was serially diluted to determine the detection limit for live virus. Analysis of RNA extracted from logarithmic dilutions indicated a detection threshold of 0.0005 TCID_50_ (10^–9^ dilution; 0.1 TCID_50_/mL; 0.0005 TCID_50_ per assay well). The threshold for detection of SARS-CoV in spiked fecal samples was 0.005 TCID_50_ (10^–7^ dilution; 1 TCID_50_/mL; 0.005 TCID_50_ per assay well) (data not shown).

**Figure 2:**
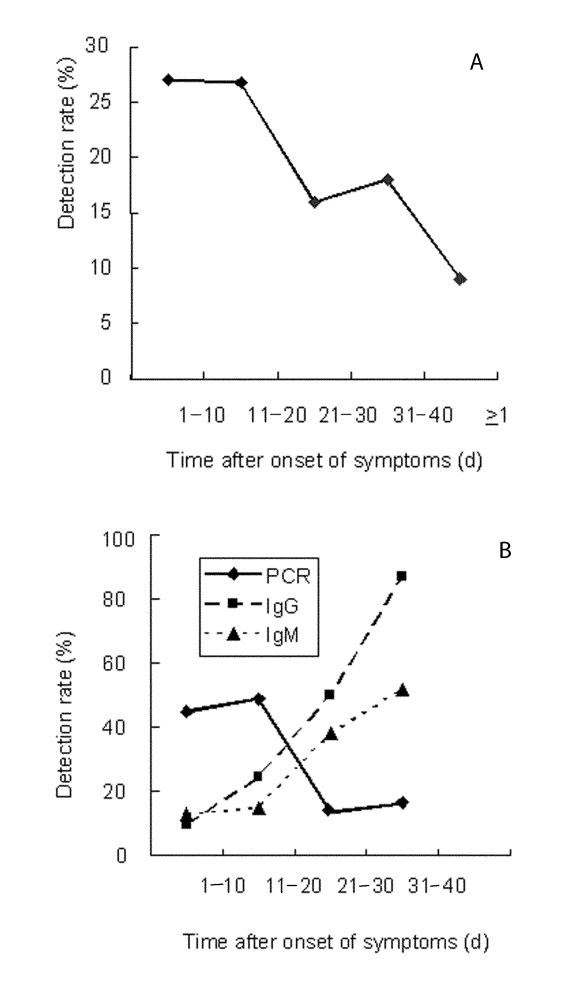
(A) real-time polymerase chain reaction (PCR) analysis of fecal samples; (B) real-time PCR, immunoglobulin (Ig) M and IgG analysis of blood samples.

Materials from persons who had probable SARS included 326 fecal samples and 426 blood samples. Control specimens collected during the outbreak from healthy persons included 16 fecal samples and 82 blood samples. The detection rate in fecal samples was 27% during the first 20 days after onset of symptoms (Table, Figure 2A). In the 20 days that followed, the detection rate declined to 16% to 18%, but even after >40 days, 9% of samples gave a positive reading. A similar time was observed in the analysis of blood samples; however, a higher the detection rate of 45% to 49% was obtained (note that only 11 of the samples were matched for blood and feces). During the first 20 days after onset of symptoms, the detection rate of RT-PCR in blood was significantly higher than that for IgM (10%–24%) or IgG antibodies (13%–15%) (Table, Figure 2B). Twenty-one to 40 days after onset of symptoms, serologic findings were more frequently positive than RT-PCR.

**Table:** Summary of clinical samples^a^

Specimens	Total patients	1–10 d		11–20 d		21–30 d		31–40 d		>40 d	
		pos	neg	pos	neg	pos	neg	pos	neg	pos	neg
Feces PCR	326	10	27	19	52	12	65	12	55	7	67
Blood PCR	426	28	34	20	21	22	143	26	132	NA	NA
Blood IgG	426	6	56	10	31	82	83	138	20	NA	NA
Blood IgM	426	8	54	6	35	63	102	82	76	NA	NA

^a^pos, positive;eg, negative; PCR, polymerase chain reaction; Ig, immunoglobulin; NA, not available.

Of the 16 fecal and 82 blood samples obtained from healthy persons, one blood sample yielded a positive result in RT-PCR (confirmed by repeated assays). Because the sample was collected during the outbreak, it may represent a true infection in a person who was not yet symptomatic or who did not have classical symptoms (no clinical information for the period after sampling was available).

We also analyzed 180 sputum and 76 throat-washing samples from an unrelated cohort of persons with a diagnosis of probable SARS, for which the time after onset of symptoms had not been reported. The RT-PCR detection rate obtained in these samples was 63% for sputa, and 15% for throat washing samples (data not shown).

It was not possible during the Beijing outbreak to obtain clinical materials in a prospective serial fashion from a defined SARS-CoV–infected patient cohort. Thus, some samples represent persons with respiratory symptoms caused by pathogens other than SARS-CoV (*10*). However, confidence in the clinical criteria is enhanced by an 87% seropositivity in samples taken 31–40 days after onset of symptoms.

Current real-time RT-PCR assays allow sensitive detection of SARS-CoV nucleic acid in clinical specimens by targeting N gene sequence, as shown here, or *pol* gene sequence (*11*–*15*). A major advantage to real-time PCR platforms is that amplification and analysis are completed in a closed system. Thus, the risk of contamination, which can confound conventional (frequently nested) RT-PCR protocols (*5*,*11*,*16*), is markedly reduced. Whether different positivity rates reported for various SARS-CoV assays (*12*–*14*,*17*) reflect true differences in assay performance, or merely differences in specimen type or differences in sample preparation (*13*), will only become apparent after comparative quality control tests using identical samples in the various assays and laboratories. Using calibrated DNA and RNA standards, we achieved comparable results with the assay reported here in the New York and Beijing laboratories.

RNA integrity is a critical determinant of sensitivity in RT-PCR SARS-CoV assays. Samples were not collected at clinical sites with the objective of nucleic acid analysis. Additionally, protocols adopted by the various hospitals for sample collection, handling, and storage were not uniform. Nonetheless, RT-PCR analysis resulted in consistent results for all 11 cases of matching feces and blood samples. Furthermore, all blood samples seropositive during the first 20 days after onset of symptoms were also positive in RT-PCR. Of the 48 RT-PCR positive samples collected 21–40 days after onset of symptoms, 45 were also seropositive.

RT-PCR analysis of blood was a less sensitive index of infection than immunologic assays at later time points (21–40 days after onset of symptoms). However, 16% of blood samples and 18% of fecal samples contained SARS-CoV RNA >31–40 days after onset of symptoms. A similar duration of persistence of SARS sequences in stool has been observed by Ren et al. (*17*). Whether infectious virus is present at these later time points remains to be determined; nonetheless, our findings indicate that long-term monitoring may be required to control dissemination of disease.
